# Supplementary material for: Epidemiological and ecological consequences of virus manipulation of host and vector in plant virus transmission
Source: PLoS Comput Biol. 2021 Dec 30;17(12):e1009759. doi: 10.1371/journal.pcbi.1009759 (PMC8754348; doi:10.1371/journal.pcbi.1009759)
Supplement: Attachment — Submitted filename: Resubmission Cover Letter.pdf [file pcbi.1009759.s007.pdf]

Dr Nik Cunniffe
Associate Professor
Department of Plant Sciences
University of Cambridge
Downing Street
Cambridge, CB2 3EA

UNIVERSITY OF CAMBRIDGE
Department of Plant Sciences

30 November, 2021

Manuscript PCOMPBIOL-D-21-01643

Epidemiological and ecological consequences of virus manipulation of host and vector in plant virus transmission

Dear Prof. Struchiner and Prof. Britton,

Thank you for handling our manuscript, and for securing such useful reviews.

Please find below our responses to the reviewers' comments.

We have also submitted an edited version of the manuscript, marking all changes via Word's "Track Changes" capability. All line numbers below are relative to the version of the manuscript as shown after selecting "All Markup" in the dropdown box on the "Tracking" section of the ribbon as shown after clicking on the "Review" menu.

Note we have also made further changes to the format of our manuscript as requested by the production staff. In particular, we have inserted an "Author Summary" (L29-44), and added an extra section providing legends for all Supporting Information (L1391-1395). We also took this opportunity of altering all references to our Supporting Information within the manuscript to what we believe is "PLOS style" (e.g. changing from "Supplementary Table S1" to "S1 Appendix", L218). Finally, the Supporting Information itself has been deleted from the main manuscript and instead put in separate files. In making this change, we also reformatted the large table in S1 Appendix to make it more digestible (apart from the single change around moving some of the comments on the paper on Shaw et al. (2017) to the Supporting Information, which are shown via track changes and mentioned in our response to Reviewer #2, all changes are entirely cosmetic, based on formatting the table more attractively).

Thank you again for handling our manuscript.

Nik Cunniffe

(on behalf of all co-authors)

Telephone: +44 1223 333954
Fax: +44 1223 333953
E-mail: njc1001@cam.ac.uk

Reviewer #1

When I review papers, I typically print them out and read them with pen in hand ready to make corrections. About halfway through the introduction, I put the pen down and simply enjoyed reading the manuscript. The manuscript is well communicated and polished. As a reviewer, I appreciate that the authors took the time to edit the manuscript to a point that it requires little more.

> **Response**. We are grateful to Reviewer #1 for engaging so positively with our work and for their complimentary comments acknowledging the care we have taken over our manuscript.
>
> **Action**. None (no changes requested/suggested by reviewer in this comment).

The focus of the manuscript is an important topic in epidemiology and has ramifications for managing the spread of insect transmitted plant pathogens. This paper is dense, and I will acknowledge in the two hours I allotted to complete my review I have not vetted each equation; doing so would take considerable time. Nonetheless, the overall approach used by the authors is appropriate and on the surface the equations and results seem reasonable and appropriate. I do encourage the authors to check their equations for typo's. It is easy to miss a subscript or that a parameter was raised to an exponent.

> **Response**. Thank you for recognizing our manuscript focuses upon "*an important topic in epidemiology*", and for checking our equations and results are reasonable and appropriate. We reassure Reviewer #1 and the Editor that we have also carefully checked our mathematics in some detail – a number of times – and are confident that no typographic errors are present.
>
> **Action**. None (no changes requested/suggested by reviewer in this comment).

I did note that there appears to be an issue with the numbering of the equations. In the "model analysis and numerical approach" section and in the "invasion threshold section" equation 26 is important. However, in my draft there is no equation 26. Rather the equation numbers skip over 26 (goes from 25 to 27). Likewise, I note that earlier in the manuscript equation 3 appears twice. Accordingly, please check the equation numbering.

> **Response**. Yes, at the very last minute before submission we inadvertently interfered with the automatic numbered of equations in MS Word at Equation (3), which caused later references to go awry. We have corrected this.
>
> **Action**. Corrected (L321). Doing this has caused Word's track changes feature to note "edits" to every Equation up to and including Equation (26) on L432, but note that each of these in fact refers simply to a change to each equation's number.

I thought the "intuitive interpretation" statement that followed equation 36 was excellent. I think a lot of readers will benefit from this.

Overall, I commend the authors for their efforts, I enjoyed reading this.

> **Response**. Again, thank you for engaging so positively with our work!
>
> **Action**. None (no changes requested/suggested by reviewer in this comment).

Reviewer #2

In this paper, Cunniffe et al describe a model that accounts for how host symptomology may impact the attractiveness of the plant to insect vectors, as well as other deviations from random uniform attraction of the insects to the hosts. They use a series of differential equation to describe their model. They found some rather cool outcomes in terms of long-term oscillations, bistability, and answers as far as the importance of vector preference for hosts.

Overall, I really like this paper. It's very elegantly addresses a lot of interesting questions all at once and handily resolves several problems being faced by plant virus epidemiologists for the past decade. Their online app is a pretty neat feature should help teachers kind of explaining portions

of plant disease epidemiology. I think that they were fairly opaque and how they described what they were parameterizing their model to. It was not clear if there was a specific disease in mind, and if there was what disease it was. I would imagine that the state parameters, transitions between states, and rate parameters would very widely between many different diseases. For instance, they don't cover if this model is supposed to focus on persistent or nonpersistent viruses, propagative viruses, or even viruses at all. In some ways this is a strength so that it gives general answers, but it left me wondering how they chose their parameter values, and how representative these parameters would be for different scenarios or different crops.

Response. We are grateful to Reviewer #2 for their very positive comments on the "*rather cool outcomes*" of our modelling work, which "*resolves several problems being faced by plant virus epidemiologists*". We were also very pleased to hear that the reviewer appreciated our online interface. We agree this is a particular strength of this work, in terms of maximizing its impact.

As the reviewer has correctly inferred, rather than parameterizing our model to focus on a single host-vector-virus pathosystem, here we were more ambitious. In particular, we sought to find "*general answers*" concerning the effect of vector preference on epidemiological dynamics. We did this by selecting baseline parameters in our model, and then examining the results of a series of very extensive parameter scans around these default parameterisations. Our results are therefore very widely applicable.

In fact we actually concentrated on a pair of sensitivity analyses, around two distinct baseline parameterisations of our model. One of these focuses on an exemplar persistently transmitted virus; the other is more appropriate for a typical non-persistently transmitted virus. We did this to search for similarities and differences between the two classes of transmission; in that sense the model is "*supposed to focus*" on both, since an explicit goal of the formulation was to handle both transmission classes in a single framework. Indeed, in the original submission we already indicated how differences between the transmission classes enter into the model equations (this description is now in the text surrounding Equations (7) and (8) between L340 and L352, as well as in Equation (27) on L434).

Indeed, differences between transmission classes are an important driver of our results as we present them. Searching for similarities and differences between transmission classes explains why the first few figures in the Results section of the manuscript consist of parallel panels – e.g. Fig 3A-C vs 3D-F, Fig 4A-C vs 4D-F, Fig 5A-C vs 5D-F – in each case focusing on non-persistent vs. persistent transmission. Our later figures are sufficiently complex that entire figures are needed to characterize model behaviour for a single transmission type, but we note that figures again come in matched pairs (e.g. Fig 6 [NPT] vs 7 [PT]; Fig 10 [PT] vs 11 [NPT]).

However, reading back the "Model analysis and numerical approach" section of the Methods in the paper as submitted, we recognize we might have said more to explain our intent and overall strategy, and have edited the paper at this point to do so.

Action. Added text L448-457.

Specific Comments:

• Line numbers and double spacing would have helped a ton for reading and reviewing

Response. Apologies for this. At this stage of the review process, and in the light of the current length of the paper, we think double-spacing would now be overkill, given that few further changes now seem likely to be required. However we have added line numbers as part of this revision in case the Editor decides to send the m/s for re-review (as well as to allow us to specify in this letter precisely where changes/edits have been made).

Action. Added line numbers.

• They rely on a constant rate of death and harvesting for the plant host and I'm not sure that that is very realistic for most scenarios. I'm taking a wild guess and saying that they did this to "keep the model well behaved" But the authors are fairly clever and may have another solution around this

Response. Assuming a constant rate of harvesting and planting is relatively common in modelling work. At the spatial scale of the model as used here, this type of assumption most closely relates to: i/ a perennial crop (for which there is constant production of susceptible host tissue); ii/ long-lived hosts such as fruit trees which can be continuously replanted; iii/ wild populations of plant host species in which there is natural turn over and recruitment except when there is stochastic environmental disturbance.

The assumption was not really made to keep the model "*well behaved*" but instead to make the mathematical analysis more tractable, given the complexity of the other aspects of the model. Indeed, we note we have gone further than we might, inasmuch as our model does allow for additional disease induced death (via our parameter μ), which has the knock-on effect of making the total size of the host plant population vary with the epidemiological state of the system. An alternative – and more common – approach would have been to make the inflow into the S compartment exactly balance the outflow due to natural/disease induced death from the S and I compartments, which would have allowed us to simplify the host epidemiology down to a single equation (since knowing I would be sufficient to immediately infer S if the fluxes were balanced in this way and so $S = N – I$). However, we discounted this, wishing to represent the disease-dependent population size that is a common feature of plant disease epidemics when host populations are subject to disease-induced death or roguing. We also note that our more careful than normal treatment of host dynamics has important consequences, including bi-stability (this was in fact treated in the Discussion of the paper as previously submitted, in the text which is now on L900-913).

A "full" treatment of host dynamics for plant disease systems – at least for crops – would almost always require moving to a semi-discrete model which distinguishes behaviour within- and between- separate growing seasons. Although this type of model is adopted sometimes, by some authors (including by members of the current authorship team in other papers), it is a class of model which is difficult to analyse. Here we therefore follow almost all modelling work in plant disease epidemiology in not doing including this here.

However, we do agree that we have made a simplification, and have acknowledged this at an appropriate point in the paper. We have also added references to the Discussion to other approaches – including our own – which would perhaps lead to a more faithful representation of the underlying biology, at the cost of – a perhaps very significant – loss of mathematical tractability.

Action. Edited text L271-273, L276-278 and L913-922.

• They seem to throw quite a bit of shade at another paper in their introduction, although it's not clear if the paper that they are criticizing is the Shaw 2017/2018 paper or the Roosien et al. (2013) paper

Response. The paper being discussed here was that by Shaw *et al.* (2017). We have edited the text to make this clearer. We have also toned down the "*shade*" by more explicitly highlighting certain of the very positive aspects of the Shaw *et al.* (2017) model formulation at this point the main text, and relegating the technical critique around the role of their parameter ξ, and how their model handles vectors losing infectivity, to footnotes in the table in S1 Appendix. Our intention in this paragraph was – of course – not to simply criticize the work of others, but instead to demark points of similarity and difference between prior work and our own, and we feel the current version of the text achieves this balance well.

Action. Edited text between L169 and L186; added footnotes to table in S1 Appendix

• “ Error! Reference source not found.” At bottom of page 15

Response. Yes, this was a side-effect of the problem with equation numbering identified by Reviewer #1, and was corrected by fixing that.

Action. No further change required (problem was fixed by the change made above).

• What are the authors parameterizing their model to? What disease is this supposed to mimic? Is it supposed to be specifically like a viral disease? Persistent? Non-persistent? I understand that they are keeping this vague but they are including quite detailed numbers in their model, a thousand plants as density for example

Response. As described above, the pair of baseline parameterizations were for a “typical” non-persistent and persistent viruses. In neither case were we aiming to mimic a single disease, but instead – by way of our extensive parameter scans – we ensured scenarios corresponding to a wide range of diseases were covered. It is not so much that we were “*keeping this vague*”, but instead the range of parameters that are potentially relevant to different pathosystems is really quite wide. For example, the Shaw *et al.* (2017) paper varies parameters over 2-3 orders of magnitude in their large global sensitivity analysis; some parameters values cited in our original source material (i.e. Madden *et al.* (2000) and Jeger *et al.* (1998)) are similarly variable. We therefore contend that our approach of extensive sensitivity analysis is in fact more valuable than the ostensibly more “realistic” treatment that would follow from targeting very precisely on a single host-virus-vector pathosystem.

However, as Reviewer #3 has noted, we nevertheless included “*detailed numbers”* in our pair of baseline parameterisations. This was unavoidable, since we wished to use a computer to perform a numerical solution of the differential equations specifying the model! In the case highlighted by the reviewer of taking the density of host plants to be N = 1,000 in the absence of disease, this was arbitrary (but also entirely unimportant, since our parameterization in fact focused on obtaining the “target” value R_0 = 2 for both classes of transmission, and so any change to N would simply be absorbed into a corresponding change to other parameters).

We also note that our online interface is quite valuable in this context, since the reader of our paper can set any or all parameters to any value they please, and examine the model’s results for themselves.

Action. Edited text L552-554 to make it clear the selected host plant density, N = 1,000, was an arbitrary – but entirely uninfluential – decision, as well as to reiterate that the online interface allows other scenarios to be explored (L568-571).

Reviewer #3

I enjoyed reading this paper, although within the relatively short space of time available for review, I couldn't claim to have really metabolized all of the information it contains. I think the authors have done a good job of packing a large quantity of work into the format of a single paper. Their aims are ambitious here, and while they achieve what they set out to do, the end result is a tightly-packed read that needs to be taken in several bites.

Response. We are grateful to Reviewer #3 for engaging with our work and for their extremely positive comments on our manuscript, as well as their careful and helpful suggestions on how the presentation might be improved.

We agree there is quite a lot to digest here – as the reviewer clearly recognises this is actually a consequence of the depth of our ambition – but we find what appears to be strong support for publication from all reviewers to be reassuring, inasmuch as it indicates we have not fallen into the trap of attempting to do too much in a single piece of work.

Action. None (no changes requested/suggested by reviewer in this comment).

The technical aspects of the paper use familiar techniques from epidemiology and mathematical biology to develop model for two different types of plant virus transmission dynamics. The mathematical approach utilizes linked differential equations and builds on a very strong lineage of research on plant virus dynamics which has been created over several decades by Prof Jeger and colleagues. This is one of the strengths of the paper. The commonality of approach used here with that existing body of work ought to facilitate comparative epidemiology, and I would have liked to see a bit more of that in the current paper. However, (as noted) there is already a lot of material to ingest and comparative work would perhaps best be saved for a purpose-written review? Still, in revision, if the authors see opportunities to highlight further any simple comparative results with previously published analyses I think I would not be alone in finding that useful.

Response. As the reviewer identifies, the paper is already rather long. We therefore did not wish to devote a huge amount of space to comparisons between different model formulations. However, we note that in the revised version of the manuscript there is a fair amount of text – most of which was present in the originally-submitted version – in which we draw comparisons between our model formulation and the past models of Gandon (2018) and – particularly – Roosien *et al.* (2013) and Shaw *et al.* (2017). This text is currently at the following locations: Introduction (L157-204), Discussion (L900-901; L907-912, L975-982; L999-1006; L1024-1031) and all of S1 Appendix.

We therefore agree with Reviewer #3 that further comparative work would be better left for a purpose written review.

Action. None.

The current introduction does a decent job of setting the scene for the technical sections of the paper, but I wondered if most of the salient information covered in the introduction could be compressed to a tabular supplement that would cross reference aspects of virus transmission biology that have been modeled with relevant literature references, without commentary on whether these previous studies were successful or not? This would give readers a quick look-up table if they are interested in reading the earlier literature for themselves, but would save considerable space in the current paper that could be allocated to a less densely packed exposition of the model.

Response. Thank you for this comment. After reflection, we decided that the current approach of introducing and commenting on what is tracked in past modelling work was important to keep in the current Introduction. We did this since we feel it acts to motivate our work, as well as to make it accessible at least in overview to the broadest possible audience, e.g. plant virologists. We have, however, and as described in our response to Reviewer #2 above, attempted to give a more balanced treatment of the past work of Shaw *et al.* (2017) (L169-193), and note that our S1 Appendix already includes the full detail of this type of comparison.

We therefore hope the Editor will condone no change in response to this comment. However, if the Editor would prefer, we are happy to revisit this suggestion in a subsequent revision.

Action. None (although we are happy to make changes if the Editor would prefer).

Building on the issue of the exposition of the model, I would encourage the authors to try to bring out the logic (by logic I mean the deterministic rules that the modeled biology is following) in the model description. The model structure diagram and other visual aids in the relevant figure are excellent, but I found the description of the model parameters and their derivation somewhat dense. The authors state that the model is complex; and it is in the sense that there are many parameters, but it has only four state variables, so in another sense it is relatively simple. Returning to the earlier point about making the exposition of the biological motivations for the model more explicit, exploiting the relative simplicity of the model in terms of the number of state variables, in order to lead the reader through the within-equation complexity could be a useful approach? One way to do this could be to label the equations in (1) (a) to (d) and then explicitly have subsection headings along the lines of "Derivation of equation 1a; the rate of change in susceptible host plants". I think

this type of approach would work well in tandem with the interface for the model which the authors have thoughtfully provided. I agree enthusiastically with them that this is an excellent way to allow readers to understand what the model does and how it reflects the underlying biology.

Response. Thank you for this comment. Again after some careful reflection – and thinking in some detail about how we could possibly re-structure the description according to this comment – we eventually determined the overwhelming driver for the description of the model appearing to be complex is because the underlying biology we capture is complex.

This remains the case even if the description of the model is structured around the individual state variables. Reviewer #3 notes the apparent simplicity our model using "only" four state variables, and suggests this might drive a simpler description of the model. However, the number of state variables is beguiling. The complexity of our model lies in how state variables affect each other, and this remains true even if material is presented in a slightly different order.

We therefore hope the Editor will condone no change in response to this comment. However, if the Editor would prefer, we are happy to revisit this suggestion in a subsequent revision.

Action. None (although we are happy to attempt these changes if the Editor would prefer).

Overall, the paper does a good job of working through the methodology and then the results. There are a few places where I think some rewording would improve the clarity. For example, the second paragraph in the section "Model equilibria" might benefit from some reworking. As currently written, I found it a bit vague for the first sentence or two. Then, when the issue of how many equilibria the model has comes up, I found the current version left me with the impression that the mathematical cart had somehow ended up in front of the biological horse. It's less interesting (at least to me) that solutions compatible with actual biology are among the possible behaviours of the model, than that the model is able to represent biology as it is known to exist; it's a subtle, but I think important, change in focus.

Response. We apologise for any lack of clarity here, and have rewritten the relevant text, hopefully bringing the horse (the biology) back to its appropriate position in front of the cart (the mathematics).

Action. Edited text L617-629.

Labeling of the vertical axes in the figures needs to be standardized. For example in figure 5C and 5F the ordinate is labeled "Host incidence". I assume this is supposed to be "Host disease incidence", but in the legend it is referred to as "Disease severity" and in other figures the same quantity (for example in Figure 9 C - F) is labeled as "Incidence" followed by the expression for the diseased fraction of the host population.

Response. Thank you for this; we agree in a complex paper it is important that the graphics and terminology are consistent, and appreciate Reviewer #3's efforts in spotting a case in which we did not ensure this. The legend was incorrect; disease severity should have read incidence. The point about including the "*expression for the diseased fraction of the host population*" on axes labels; we included this whenever we specifically refer to the terminal incidence (i.e. as $t \rightarrow \infty$) [and so is the case in Figures 5A and 5D, as well as the parts of Figure 9 referred to by Reviewer #3]. We have made this clearer by editing the labelling of the axes in the figure itself, and in particular to note that the axes in Figures 5B, 5C, 5E and 5F refer to either the initial (Figures 5B and 5E) or the time-dependent (Figures 5C and 5F) values of these quantities. We considered editing the y-axes labels for Figures 5A and 5D to emphasise that the (equilibrium) host disease incidence is being shown, but decided not to in order to ensure the labels in this figure were consistent with those in other parts of the paper (including Figure 9 as identified by Reviewer #3). In carefully checking all axes labels, we also noticed that those on Figures 11E and 11F were misleading, inasmuch as the graph was not showing a terminal incidence but

rather a time dependent one. This was obvious from context, but nevertheless we have corrected this.

Action. Edited legend to Figure 5 (L675 and L683), as well as altered the y-axis labels on Figures 5B, 5C, 5E and 5F and Figures 11E and 11F.

Figure 9 contains another element of the paper that (I think) requires a bit of attention. If I have followed the analysis presented, the model contains a section of parameter space in which it illustrates bistability in the absence of the vector. Solutions with this property apparently exist for both PT and NPT types. Since this is, when taken at face value, a surprising outcome, the authors devote some space to offering explanations. It could be an illustration of my lack of comprehension, but I think the story is still missing something. Is the result to be interpreted as being time-bounded in some way? I ask, because, without the presence of a vector I can't think of a biological mechanism through which a vectored virus could be transmitted from infected plants to healthy ones, and unless that happens, won't the virus be eliminated from the system, eventually when the infected plants are removed (by virus-induced mortality, roguing, or harvest?). In other words, in a system with no vector and a composite removal rate greater than zero, doesn't the infected host represent a dynamical dead-end for the virus?

Response. Apologies, we see that the caption of Figure 9 is not entirely clear, and this might have led to this confusion. “Bistability (no vector)” does not mean the vector is not present, but instead that in the *absence of virus* the vector would die out, with the virus “rescuing” the vector by causing infected plants to act as better habitat for vectors (thereby allowing both vector and virus to persist at equilibrium). The referee is entirely correct that if the vector were to die out, then the virus would have to die out too, at least in the long term. But that is not happening here. We have made edits to make this important point more obvious.

Action. Edited caption and legend to the Figure to make this clearer (L802-804)

In the section "Ecological context" I think the first paragraph needs some attention. The writing in that paragraph in particular reads as though it was written rather quickly and there are a couple of places where the grammar is questionable. The overall result is that it's not clear (at least to me) what the point is/was.

Response. Thank you for these comments. We agree that the first paragraph was rather unclear concerning the points being made, and in setting the scene for what followed. We were highlighting that field-based studies to determine the epidemiological significance of vector preference given the broader ecological context may provide difficult, and so will require innovative solutions.

Action. Edited text L1051-1064 to make both this text and the purpose of the following paragraphs more clear.

The second half of the same section (from the final paragraph on the 38th page of the pdf file down to the start of the section "Evolutionary implications" seems mostly irrelevant to the main points of the paper. It deals with contextual results gathered in other studies, but adds little to the explanation of the key findings of the current work. I would recommend the authors remove this section and use the space saved to give a clearer account of the importance of the results from the new model.

Response. Respectfully, as we have now hopefully set out more successfully in the preceding paragraph, in our view it will be essential to consider the broader ecological context. This will affect not only future research needs, but also interpretation of modelling results. We have identified several examples and highlighted – for each – future research that we feel is necessary (each paragraph ends with this identification).

Action. Various minor edits to text L1065-1122, to sharpen language and to ensure each paragraph ends with a clear prescription for future research.

Summing up, the paper is an important and timely contribution to the literature on plant virus dynamics. The focus on mechanisms that are studied by empirical biologists in the field and lab should facilitate the interaction between modelers and experimenters in future and the authors deserve a warm thank you from their peers for providing a template for how such detailed modeling can be done. Indeed, the authors make rather little of the conceptual/methodological contribution that the paper makes.

Response. Thank you for these very positive comments on our work! However, if it is acceptable to the Editor, we would prefer to not include text in the paper highlighting what we feel are our conceptual and/or methodological contributions. Instead we would simply prefer to allow the reader to come to a decision on the quality of our paper for themselves.

Action. None (although we are happy to make changes in response to this comment if the Editor would prefer).

Reviewer #4

Cunniffe and colleagues have tackled a very important problem: the epidemiological consequences of virus manipulation of the host and vector. Most plant viruses are transmitted by insects (primarily Hemiptera) or other arthropods, and these viruses can generally be classified into two broad categories (nonpersistent and persistent) in terms of their transmission properties, or four categories (nonpersistent, semi-persistent, persistent-nonpropagative, and persistent-propagative). The large epidemiological consequences of these categories were previously studied using theoretical SEIR models for the virus and the vector. But these past studies generally did not consider host and vector manipulation by the virus and many other complexities of the pathosystems. For instance, the infection status of a plant can affect the feeding behavior of the vector, virus-status of the vector can affect its behavior. Cunniffe et al. have greatly extended past theoretical work by developing an elaborate deterministic SI compartmental differential equation model for the system, with components for the plant and vector. Emphasis is on how epidemic results differ for nonpersistent and persistent categories.

Response. We are grateful to Reviewer #4 for their useful comments which put our work in context, and for their helpful suggestions below.

Action. None (no changes requested/suggested by reviewer in this comment).

For the most part, the authors have clearly described the model terms (variables and parameters [and there are MANY parameters]), and have shown detailed results for the nonpersistent and persistent transmission cases. Besides the extensive simulation results, they derived model equilibria and the important basic reproduction number, R0. The results make sense and advance our knowledge of plant virus epidemiology. The work deserves publication.

Response. Reviewer #4 is correct that there are many parameters, but I think they also acknowledge that this complexity is an unavoidable consequence of the complexity of the biology we model.

We also very much appreciate the reviewer's strong and unambiguous support that our work should be published.

Action. None (no changes requested/suggested by reviewer in this comment).

This is an EXTREMELY long and detailed manuscript. It would be impossible for me to check all of their mathematical results in the allotted time for review. In fact, it would probably take me a year to confirm all of their derivations, especially those presented in the appendices. However, based on past work, I have confidence in the mathematical work by these authors.

Response. Again, the length of the technical part of the manuscript (i.e. the Methods and Results) is a consequence of the ambition, but we are grateful that the reviewer has confidence

in our work. We reassure both Reviewer #4 and the Editor that we have carefully checked our derivations, and so we also have full confidence in our mathematical work.

Action. None (no changes requested/suggested by reviewer in this comment).

Despite the complexity of the model (at least in terms of number of parameters), the authors have made some major simplifications. This is always done with theoretical modeling, of course. But I think the authors should add some discussion to justify these simplifications, and perhaps speculate some more on how their results might be sensitive to the simplifications. This could inspire future work. (The authors have already discussed other complexities of these pathosystems not covered by their model). Given the length of the current manuscript, and the many important results, expansion of the model would only be appropriate for future research and future papers (in my view).

Response. Thank you for this comment, and in particular the explicit comment that expansion of the model would only be appropriate for future research/papers (i.e. that Reviewer #4 agrees it is acceptable not to attempt to include new areas in the current m/s).

Action. None (no changes requested/suggested by reviewer in this comment).

To start with, the authors are using an SI formulation, ignoring the latent (E) and removed (R) states in the plant and vector populations. This is a very common thing to do, especially because it facilitates the calculation of equilibria (steady states, and so on). With this approach, however, removed plants are replaced immediately by new healthy plants. This may be reasonable for epidemics of trees over many years, but with annual crops (wheat infected by barley yellows), removed/dead plants are never replaced within the epidemic. Surely, this merits some discussion.

Response. The potential effects of two model simplifications are combined in the above comment. As Reviewer #4 acknowledges, ignoring the latent class (i.e. going from what would be called an SI to an SEI model in the wider mathematical biology literature) is relatively common in plant disease epidemiology, mainly to facilitate analysis. In general the impacts of this simplification are well understood, although the interaction here of the density of exposed plants with probabilistic choices made by vectors means there would still be unanswered questions. We have added some text on this to the Discussion.

The second critique is that – in some systems - plants that are rogued out due to disease are not necessarily replanted. This is true, although we note again that we have gone beyond the simplest treatment – as adopted by other authors such as Roosien and Shaw – which assumes the host population density is constant. We agree that host plants are often not rogued in seasonal cropping systems, although we see a more careful treatment of roguing as being an additional factor which could potentially be added were we to attempt a more careful treatment of host dynamics, and have added some text on this to the Discussion.

Action. Added text to Discussion (L923-931 [on latent periods in plants] and L918-922 [on roguing])

The authors are grouping three categories of transmission (semi-persistent, persistent-nonpropagative, persistent-propagative) into one broad category. Viruses in their nonpersistent category vary a lot in terms of acquisition and inoculation rates, latent period in the vector, and so on. Is it really reasonable to make conclusions about results for this broad category? I am sure that varying model parameters could handle the different (sub-)categories, except for the latent period in the vector (since an E category for the vector is not considered).

Response. We agree; we simplified in the name of analytic tractability, but should acknowledge this. We now do in the Discussion.

Action. Added text to Discussion (L931-937 [on latent periods in the vector and the differences between semi-persistent vs. persistent non-propagative vs. persistent propagative]).

Many epidemics by nonpersistent plant viruses are driven by immigrating/emigrating vectors. For example, aphids from other fields/weeds fly into a soybean field, feed for a few minutes and transmit soybean mosaic virus, and then fly away to another field. Yet, emigration and immigration are not considered in their model. Surely, this also merits some discussion (at least for future work).

Response. Again, although the "original" models of vector preference (e.g. Jeger et al. (1998), Madden et al. (2000)) included immigration and emigration, omitting it is a very common simplification in this type of modelling work. We note our parameter δ in fact can be interpreted as accounting for emigration – and even allows the rate at which emigration occurs to depend on the current infection status of the host population – migration is entirely omitted. In part this is because as soon as any non-zero proportion of viruliferous migrants are accounted for, then disease is always able to invade.

However, we agree that this is an important point, particularly for non-persistently transmitted viruses. We therefore now address this in the Discussion. This includes a reference to a preprint based on the recent work of one of us which focuses on how the interplay between migrant and colonizing aphids might be modelled. Since that work does not include vector preference, an obvious extension for the future is to link these two modelling frameworks.

Action. Added text to Discussion (L938-949).
