# Supplementary material for: Epidemiological and ecological consequences of virus manipulation of host and vector in plant virus transmission
Source: PLoS Comput Biol. 2021 Dec 30;17(12):e1009759. doi: 10.1371/journal.pcbi.1009759 (PMC8754348; doi:10.1371/journal.pcbi.1009759)
Supplement: Attachment — Submitted filename: Review of Cuniffe.docx [file pcbi.1009759.s005.docx]

Review of Cunniffe

In this paper, Cunniffe et al describe a model that accounts for how host symptomology may impact the attractiveness of the plant to insect vectors, as well as other deviations from random uniform attraction of the insects to the hosts. They use a series of differential equation to describe their model. They found some rather cool outcomes in terms of long-term oscillations, bistability, and answers as far as the importance of vector preference for hosts.

Overall, I really like this paper. It's very elegantly addresses a lot of interesting questions all at once and handily resolves several problems being faced by plant virus epidemiologists for the past decade. Their online app is a pretty neat feature should help teachers kind of explaining portions of plant disease epidemiology. I think that they were fairly opaque and how they described what they were parameterizing their model to. It was not clear if there was a specific disease in mind, and if there was what disease it was. I would imagine that the state parameters, transitions between states, and rate parameters would very widely between many different diseases. For instance, they don't cover if this model is supposed to focus on persistent or non-persistent viruses, propagative viruses, or even viruses at all. In some ways this is a strength so that it gives general answers, but it left me wondering how they chose their parameter values, and how representative these parameters would be for different scenarios or different crops.

Specific Comments:

- Line numbers and double spacing would have helped a ton for reading and reviewing
- They rely on a constant rate of death and harvesting for the plant host and I'm not sure that that is very realistic for most scenarios. I'm taking a wild guess and saying that they did this to “keep the model well behaved” But the authors are fairly clever and may have another solution around this
- They seem to throw quite a bit of shade at another paper in their introduction, although it's not clear if the paper that they are criticizing is the Shaw 2017/2018 paper or the Roosien et al. (2013) paper
- “ Error! Reference source not found.” At bottom of page 15
- What are the authors parameterizing their model to? What disease is this supposed to mimic? Is it supposed to be specifically like a viral disease? Persistent? Non-persistent? I understand that they are keeping this vague but they are including quite detailed numbers in their model, a thousand plants as density for example
